# Who continued travelling by bus in different periods of COVID-19? A data-driven analysis from Shanghai, China

**DOI:** 10.1371/journal.pone.0328700

**Published:** 2025-09-03

**Authors:** Weihan Bi, Yu Shen, Yuxiong Ji, Yuchuan Du

**Affiliations:** Key Laboratory of Road and Traffic Engineering of the Ministry of Education, Tongji University, Shanghai, China; Chang’an University, CHINA

## Abstract

The COVID-19 pandemic has caused unprecedented disruptions to individual travel behavior in the public transport (PT) system. To attract more travelers to use PT in the post-pandemic era, it is necessary to understand the heterogeneity of travel behavior changes among various user segments and across different pandemic periods. This paper presents a two-step analysis framework for discovering the inter-segment and intra-segment changes in travel patterns of PT users based on ticketing data. The framework is then applied to the case of Jiading bus transit in Shanghai, China during the COVID-19 pandemic in 2022. The results suggest that commuters are more vulnerable to the impacts of the pandemic than non-commuters, with a considerable tendency to give up commuting by bus or even permanently discontinue bus usage after the lockdown in Shanghai. Only 17.7% of bus users maintain their travel patterns after the lockdown, with a few observable sub-segments of users comprising students and employees. The findings in this study aid PT operators in achieving a better recovery in the post-pandemic era and enhance their preparedness to tackle challenges in demand management for potential crises.

## Introduction

The COVID-19 pandemic represents the most severe global public health emergency since the 1918 influenza outbreak. In response, governments worldwide implemented stringent containment measures, empirically demonstrated to effectively mitigate viral transmission [1]. While these policies preserved public health and social stability, they concurrently caused substantial disruptions to the public transport (PT) system. Mobility restriction policies coupled with public safety concerns led to a drastic reduction in PT ridership, reaching a peak decline of almost 90% during the most severe periods of the pandemic [2].

The ridership decline imposed significant financial burdens on PT agencies, placing high pressure on governmental compensation efforts [3]. An extreme case is that over one-third of bus routes in Baoding, China were suspended due to the financial strain on the operator after the pandemic. Hence, in the post-pandemic era, it holds immense importance to redesign and optimize PT services to align with the shifting demand patterns, which in turn will entice a broader audience to opt for PT once again.

To optimize PT system operations, a thorough understanding of user travel behavior is crucial. Analyzing temporal travel patterns, for example, enables operators to adjust service frequencies effectively [4], while examining spatial trip distributions informs decisions on stopping patterns and short-turn strategies, ensuring services better align with demand and enhance service quality [5]. Additionally, user attributes play a key role in service planning. Different user groups exhibit distinct priorities: low-income travelers may prioritize affordability, whereas business commuters likely value punctuality and comfort [6]. Recognizing these diverse needs allows operators to tailor services more effectively, improving overall user satisfaction.

Existing research has extensively examined travel behavior during the COVID-19 pandemic, with many studies focusing on aggregate-level trends, such as system-wide ridership declines. [7–9]. While some studies have examined behavioral differences among user groups, most adopt sociodemographic classifications instead of segmenting users by travel patterns [10–12]. This approach faces inherent limitations, as sociodemographic data are often unavailable due to privacy protections and cost constraints. Consequently, deriving user groups directly from travel records emerges as a critical alternative, which not only reveals essential behavioral characteristics but also enables the inference of user attributes from behavioral patterns, offering deeper insights into pandemic-induced behavioral shifts [13]. However, behavior-based segmentation and its analytical applications remain underdeveloped in public transport research, especially in pandemic contexts.

The passive travel data collected during various phases of the COVID-19 pandemic, particularly smart card ticketing records, has provided a unique opportunity to examine heterogeneous travel patterns among PT users. Utilizing 14 months of bus ticketing data, this study develops a two-step analytical framework to identify individuals who either changed or maintained their patterns following the lockdown, and characterize typical user groups by incorporating policy response knowledge. The study specifically addresses two key research questions corresponding to these analytical steps.

1. How did PT users exhibit distinct travel pattern adaptations during different pandemic periods?
2. What attributes were associated with PT users exhibiting consistent travel patterns during the pandemic?

To answer the two questions, we employed a data-driven approach using the Jiading bus system in Shanghai, China as a case study, which offers unique insights due to its exposure to diverse COVID-19 policy responses, including the stringent ‘Static Management’ lockdown under China’s Zero-COVID strategy. Our analytical framework proceeds in two stages. First, we classified bus users into distinct travel pattern groups based on their behavioral characteristics during two near-normal periods before and after the March 2022 outbreak. By comparing the segments a user belongs to in the two periods, we identified those who changed or maintained their travel patterns, revealing the outbreak’s behavioral impacts. Second, focusing specifically on those who maintained their travel patterns, we performed secondary clustering based on their daily travel profiles. Leveraging the differential timing of policy impacts across population subgroups, we inferred about user attributes (particularly occupation and transit dependence) through spatiotemporal analysis of trip distribution patterns within each cluster.

The remainder of this paper is organized as follows. The Literature review section provides a review on how travel behavior and patterns are affected by the COVID-19 pandemic. The Materials section introduces the research materials, including the data and study case. The Methods section presents the methodology applied to this study, followed by the presentation and discussion of results in the Results section. The Conclusions section summarizes the conclusions and offers the policy implications of this study.

## Literature review

Since the onset of the COVID-19 pandemic, numerous studies worldwide have examined and modeled how travelers’ behavior and travel patterns are affected by the crisis, revealing both diverse and common trends across different nations.

One of the most immediate and observable impacts of the pandemic on travel behavior is the substantial reduction in overall travel volume. For instance, according to Wang and Noland [14], ridership on New York City’s bike-share and subway systems declined by 70% and 95% respectively. Similarly, Jenelius and Cebecauer [15] reported a 40%–60% decrease in PT ridership across Sweden’s three most populous regions during spring 2020. Although the degree of reduction varies across travel modes and regions, the overall decline in demand is stable across cases, primarily driven by the extreme and complex policy measures implemented in response to the pandemic [16].

The travel patterns of individuals are also changed by the pandemic in terms of mode choice, travel purpose, spatial/temporal patterns, and so on. Due to concerns about virus transmission in crowded and enclosed environments, people are shifting to a large extent from shared mobility to private mobility [17]. According to the Netherlands Mobility Panel data, a substantial proportion of individuals anticipate reducing their usage of PT, whereas more people expect to increase their usage when it comes to private cars [18]. As for the spatial patterns, network modeling reveals that travelers in New York City, US tend to form smaller, tightly interconnected communities with heightened local connectivity after the pandemic [19].

Regarding travel purposes, individuals are inclined to curtail less important trips but remain obliged to travel for essential purposes during the pandemic. A statistical analysis by Abdullah *et al*. in Pakistan indicated that fewer people traveled for work and study during the pandemic as compared to the before-pandemic situation, while more people traveled for shopping [20]. Likewise, Politis *et al*. witnessed a significant decline in Greece in terms of commute trips, although a notable proportion of individuals continued to commute throughout the pandemic [21].

The shifts in travel purposes, to some extent, account for the observed variations in travel duration and spatial mobility patterns. Li *et al*. discovered the disappearance of weekday morning and evening peaks in taxi trips during the first two waves of the COVID-19 outbreak in New York City, US, which resulted from the decreasing commuter demand due to the work-from-home requirements [22]. Arias-Molinares *et al*. found that micro-mobility trips in residential and commercial areas gained greater prominence compared to others associated with work, study, leisure, or cultural destinations in Madrid, Spain [23]. Global Positioning System (GPS) tracking data from Switzerland reveals that work-from-home arrangements have significantly altered the distribution of trip purposes, subsequently reducing overall travel demand. These changes manifest in shorter average trip distances, decreased travel durations, lower trip frequencies, and diminished morning peak-hour traffic volumes[24].

The above studies have revealed and modeled the impacts of the pandemic on travel behavior, offering valuable insights to tackle the challenges faced by the transportation system during the pandemic. However, most of the studies aim at a certain region or a specific transportation system, thus the object is all travelers within the region/system, making it difficult to investigate the diversity of travel behavior in various user segments. Typically, the user segments whose travel behavior remained consistent during the pandemic have not received sufficient attention.

To the best of our knowledge, existing studies have examined the pandemic’s heterogeneous impacts across traveler groups, typically segmenting individuals by socioeconomic attributes and analyzing travel patterns within each segment. For example, Ruiz-Euler *et al*. discovered that the decline in mobility was faster for the rich and slower for the poor in the first quarter of 2020 in US urban areas [25], reflecting a widespread mobility gap during the pandemic. Similar studies also include Almlof *et al*. in Stockholm, Sweden [10], Kar *et al*. in Ohio, US [12], and so on.

However, socioeconomic attributes are often unavailable due to privacy concerns and data acquisition costs, necessitating the direct extraction of traveler segments from passive travel data sources including GPS records, smart card transactions, and mobile signal data. This type of study includes Choi *et al*. who examined the travel patterns of free-floating e-bike sharing users in Seoul, South Korea during the pandemic using the riding trip data [26], and Lizana *et al*. who investigated the PT usage change in Santiago, Chile between two pandemic phrases with smart card data [27]. A study closely related to our work is that of Lin *et al*., which similarly examines pandemic-induced shifts in individual-level bus usage patterns, using ticketing data from Jeju, Korea [28]. While their research effectively pinpoints the timing, direction, and duration of behavioral changes, their definition of travel patterns remains somewhat rough, and their analysis does not explore the user attributes underlying each distinct behavioral change pattern. In conclusion, the data-driven research on behavior-based traveler segmentation is still limited in research questions and scenarios. More efforts are needed to extend this method to other cases with different pandemic situations, policy responses, and transportation systems.

## Materials

We used the local bus transit network in Jiading, Shanghai as a case study. Jiading is a district located northwest of Shanghai with an area of 463.9 square kilometers and 1,893,400 permanent residents. Bus transit is the primary mode of PT in Jiading, accounting for 36% of motorized transportation use within the district. Shanghai Jiading Public Transport Company. is the primary operator of bus transit in Jiading. It manages a comprehensive bus transit network covering a total distance of 1,706km, operating 96 routes and serving 998 stops. This extensive network caters to more than 90% of the population in the district.

### Bus ticketing dataset

The longitudinal ticketing data utilized in this study is collected from the Jiading bus automatic fare collection (AFC) system, encompassing both smart card and quick response (QR) code data. Currently, over 84% of trips are validated through smart cards or QR codes, ensuring a robust representation for analyzing bus user travel behavior. This study assumes that each user possesses only one smart card or QR code account, allowing us to use the smart card or QR code ID as the user ID. Jiading bus operates an entry-only AFC system, meaning passengers swipe their smart card or QR code only upon boarding. Consequently, each ticketing record includes the user ID, boarding timestamp, and bus route.

The dataset comprises ticketing data from September 2021 to December 2022, covering both the pre-Omicron and Omicron eras in mainland China. After cleaning the data to remove duplicates and errors, the final dataset contains 27,935,255 records from 3,049,578 distinct users.

### The COVID-19 pandemic in Shanghai

As China’s largest city, Shanghai represents a particularly instructive case study due to its unique pandemic experience. The city not only recorded the nation’s highest COVID-19 case numbers but also implemented the most extensive and stringent lockdown measures. Unlike the localized, time-limited restrictions adopted in other countries and regions, Shanghai’s three-month citywide lockdown completely suspended all travel activities. This unprecedented scale and duration of containment measures may yield distinctive patterns in post-lockdown travel behavior that diverge from established findings in existing literature.

Before the emergence of the Omicron variant, the pandemic was effectively managed in Shanghai under the ‘Dynamic Zero-COVID’ strategy. Authorities took a relatively lenient approach compared to other Chinese cities, maintaining a near-normal order of work and life. However, the highly transmissible Omicron variant diminished the effectiveness of this approach, leading to an outbreak that began in March 2022. Despite immediate stricter control measures, the spread of the virus could not be contained. As China continued to adhere to the Zero-COVID strategy, Shanghai underwent a citywide lockdown starting April 1, 2022. After confirmed cases slowed to zero, the lockdown was lifted on June 1, 2022. Shanghai then entered a three-month phase of resuming work and production, returning to near-normal operations by October 2022.

**Fig 1:**
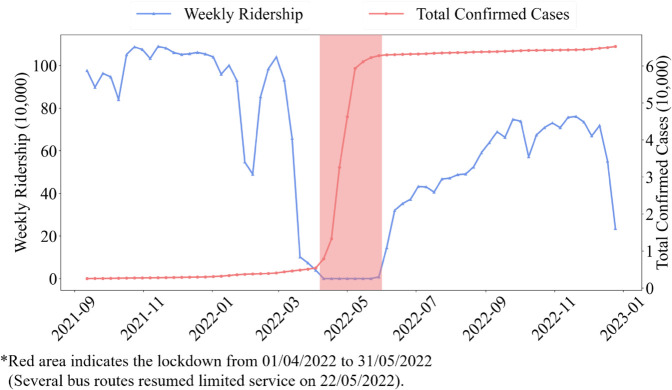
Weekly ridership of Jiading bus and total confirmed cases.

The pandemic and associated policy responses significantly impacted bus ridership, causing substantial fluctuations throughout different periods. Fig 1 depicts the weekly ridership in the Jiading bus system as well as the total confirmed cases during the 69 weeks covered by the ticketing data. During the pre-lockdown near-normal period (prior to March 2022), the system maintained a stable weekly ridership of approximately 1 million passenger trips. The subsequent outbreak and implementation of strict citywide lockdown measures resulted in an immediate and complete cessation of ridership. In the post-lockdown recovery phase (October 2022 onward), ridership demonstrated a gradual rebound, ultimately stabilizing at approximately 70% of pre-pandemic levels. The observed recovery level falls notably below that of comparable bus transit systems in regions with less stringent restrictions, including Richmond, USA (93%) [29] and Seoul, Korea (80%) [30]. This pronounced disparity underscores the particularly severe impact of pandemic containment measures on PT demand in our study context. Such marked suppression of ridership recovery offers unique insights for examining structural shifts in travel patterns under extreme disruption scenarios.

A noteworthy phenomenon during the pandemic is the distinct pandemic control measures for various populations at different times. This temporal variation in control measures offers insights for understanding user attributes through passive travel data analysis. Before the citywide lockdown on April 1, 2022, primary and secondary schools transitioned to online classes on March 12, 2022. This change meant students no longer needed to commute after this date. Thus, if a bus user ceased traveling immediately following March 12, they are likely a student. When PT partially resumed on May 22, 2022, it was limited to ‘essential workers’ employed by designated enterprises. The general public could only travel starting June 1, 2022. Consequently, bus users who resumed travel as early as May 22 can likely be categorized as ‘essential workers’. The upcoming sections will explore how this information aids in analyzing user attributes.

### Choice of analysis periods and subjects

Three analysis periods are chosen in this study to represent the typical situations characterized by the pandemic trends and policy responses during the pandemic:

1. October 11, 2021–December 5, 2021: ‘Pre-outbreak’ period;
2. March 1, 2022–August 31, 2022: ‘Outbreak’ period;
3. October 10, 2022–December 3, 2022: ‘Post-outbreak’ period.

The ‘pre-outbreak’ and ‘post-outbreak’ periods are referred to together as the two ‘near-normal’ periods, as the pandemic situations and policy responses in the two periods allowed the normal order of work and life in most areas in Shanghai. Both periods are 8 weeks in length, not affected by public holidays, and located in similar months of the year, ensuring comparability and ruling out the influence of non-pandemic factors like seasonal patterns. The outbreak period contains the period from the emergence of the index case to the full restoration to near-normal order (when the ridership recovery plateaued out). In this way, it completely tracks the behavior of bus users exiting and returning to the Jiading bus system during the whole outbreak process.

**Fig 2:**
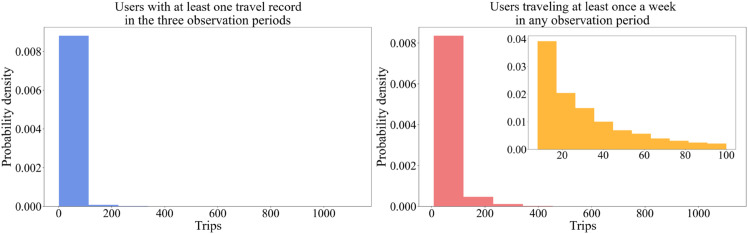
Probability density distribution of bus users with different trip records.

In the longitudinal ticketing dataset, a considerable share of bus users has only traveled once or twice, some even have never traveled in the analysis periods. These users are not relevant to the analysis and are thus excluded. Only those who have traveled at least once a week on average in either of the three periods are considered the subjects of this study. Fig 2 indicates that the Jiading bus is a PT system full of low-frequency users. 2,639,379 users have at least one trip record in the three periods, but only 400,000 users have traveled at least once a week on average in any period, who are qualified for the subject of this study.

## Methods

To systematically understand the heterogeneous impacts of the COVID-19 pandemic on the behavior of bus users, a two-step analysis framework based on bus ticketing data is proposed. In the absence of sociodemographic data, we developed a two-step analytical framework leveraging bus ticketing data to classify users into distinct groups according to their travel patterns. A travel pattern, in this context, represents a unique cluster of travel behavior defined by a combination of multiple behavioral features. For example, A group of bus users who share high route stability, frequent weekday peak-hour trips, and minimal weekend travel can form a travel pattern, distinguishing them from sporadic riders with irregular routes and schedules. While clustering may not precisely predict individual user attributes, it remains highly effective at the group level for uncovering both inter- and intra-segment variations in public transit travel behavior across different phases of the pandemic.

In the first step, a primary user segmentation is performed according to the travel patterns in the near-normal periods. The travel patterns are represented by several typical features describing the travel behavior of users over a certain period. The user segments obtained from this step can reflect the basic travel patterns of bus users with almost no interference of pandemic factors. Subsequently, comparing the the basic travel patterns of the same group of users at different periods can provide insights on the impacts of key policy responses such as the lockdown.

In the second step, we further segmented the typical user groups following the day-to-day travel profiles during the outbreak period. The day-to-day travel profile is defined as a vector that records the number of daily trips over a certain period. It possesses rich information about bus users such as travel frequency and the timing of exiting and returning to the bus system. The segmentation in this step enables a direct understanding of the various individual responses during the outbreak and allows for the inference of user attributes. Then the temporal and spatial patterns are investigated to further provide evidence for the inference results.

**Fig 3:**
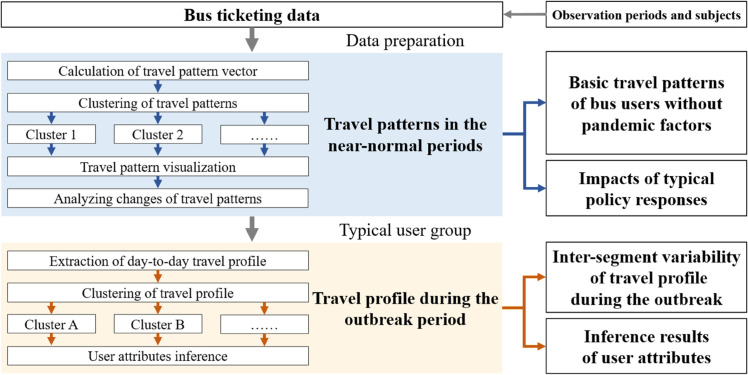
An overview of the two-step analysis framework.

The methodology framework in this study is shown in Fig 3.

### Analysis of travel patterns in the near-normal periods

The first user segmentation in the analysis framework is conducted in the near-normal periods through K-means++ clustering. The near-normal periods can be approximately regarded as COVID-free and thus present the basic travel patterns without pandemic factors. In addition, the two near-normal periods in 2021 and 2022 are similar in the months of the year, with the only variable being before/after the 2022 lockdown. Therefore, comparing the change of behavior illustrates the impacts of lockdown on the travel behavior patterns of bus users.

The feature vector used for clustering is inspired by Eltved *et al*. [31], but has been further refined and adapted to suit our specific scenario. Eltved *et al*. focused on operational disruptions on a single line and considered only travel regularity, intensity, and whether trips occurred on weekdays or weekends. However, their approach lacks spatial information and is insufficient for network-level analysis, such as in our Jiading bus case. To account for both trip frequency and the temporal and spatial regularity of travel behavior, each feature vector $V_{i}^{p}$ consists of five features, where *i* represents the unique user ID and p∈{2021, 2022} refers to the specific near-normal period. The details and descriptions of the proposed feature vector are provided in Table 1.

**Table 1:** Cluster features to describe travel behavior patterns.

Feature	Domain	Description
*Active weeks*	1–8	The number of weeks with at least one trip during the 8 weeks.
*Active days per active week*	1–7	The average number of days with at least one trip in each active week.
*Weekday peak-hour trip share*	0–1	Share of trips made in the peak hours (7–9, 17–19) of weekdays (Monday–Friday) during the whole period.
*Weekend trip share*	0–1	Share of trips made on weekends (Saturday–Sunday) during the whole period.
*Route stability*	0–1	Share of trips made on the route that was most frequently used by the user in the whole period.

The first two features—*Active weeks* and *Active days per active week* describe the frequency and regularity of travel. *Weekday peak-hour trip share* and *Weekend trip share* illustrate the temporal distribution of trips. In the absence of boarding and alighting stop data, we used *Route Stability* to provide a general measure of spatial stability at the route level. While a user may travel between different stops on the same route, this still represents a more stable spatial pattern compared to traveling on multiple routes, and thus remains a meaningful indicator. To fairly compare users’ travel behavior patterns before and after the lockdown, the feature vectors in the two periods are clustered all together. The clusters containing $V_{i}^{2021}$ and $V_{i}^{2022}$ represent the segments of travel behavior patterns that user *i* belongs to in the respective near-normal period.

Accordingly, the five features are expected to effectively segment all bus users into various groups with distinct travel patterns that correspond to different trip purposes. For instance, users who primarily rely on buses for work or school trips are expected to display highly regular behavior across the study periods—traveling with predictable weekly patterns, predominantly during weekday peak hours rather than weekends, and often using the same route. In contrast, those using buses for purposes such as shopping, running business errands, or maintaining social contacts are expected to display less regular travel patterns, with fewer trips during weekday peak hours and a lower likelihood of following a fixed route. These fundamental patterns of human activity form the basis for inferring user attributes in our study.

The K-means++ clustering algorithm is employed to cluster feature vectors during near-normal periods. This method has been widely used to uncover travel behavior patterns in transportation systems, as demonstrated by Ma *et al*. [32] and Xing el al. [33]. K-means++ is an enhanced version of the traditional K-means algorithm, marked by its probabilistic selection of initial cluster centers [34]. The first cluster center is randomly chosen from the data points, while each subsequent center is selected from the remaining points with a probability proportional to the squared distance from the nearest existing center. This approach helps K-means++ avoid the super-polynomial worst-case runtime and poor approximations typical of standard K-means. Additionally, K-means++ has a computational complexity of only O(logk), with *k* being the number of clusters, making it efficient for processing large numbers of bus user feature vectors.

As in previous studies, we used Euclidean distance as the dissimilarity measure between vectors, given that the features are continuous and geometrically interpretable. All features are standardized to ensure equal contribution to the clustering process and to prevent bias. The optimal number of clusters, *k*, is determined using the elbow method and validated with the Silhouette score. The elbow point, where adding more clusters no longer significantly reduces the within-cluster sum of squared distances, indicates the best value for *k*. A higher Silhouette score at this point confirms the accuracy of the chosen *k*.

Finally, a Sankey diagram is generated based on segmentation results from the two near-normal periods to visually track changes in user travel patterns. The rectangles in the diagram represent user segments, while the arcs illustrate the flow of users between 2021 and 2022 after the lockdown. This enables quantification of the lockdown’s post-impact on travel behavior.

### Analysis of day-to-day travel profiles in the outbreak period

The second user segmentation aims to explore the inter-segment variability of travel behavior during the pandemic and to infer user attributes. In this analysis, we particularly focused on the exit and return characteristics during the outbreak. By aligning these characteristics with the timing of policies targeting different population groups, the attributes of user segments—including occupation and dependence on bus transit—can be inferred and further validated through the temporal and spatial distribution of trips.

Inspired by Egu and Bonnel [35], we used the number of trips per day as the feature vector, which directly captures the longitudinal changes in user travel behavior, highlighting when users exit and return to the bus system. Let there be *D* days in the outbreak period, the feature vector can be expressed as $N_{i}=[n_{i}^{1},\cdots ,n_{i}^{k},\cdots ,n_{i}^{D}]$, where $n_{i}^{k}$ signals the number of trips conducted by user *i* on day *k*. Since this feature vector comprises a sequence of continuous, real-valued elements, it can be treated as a time series, making the user segmentation in this section a time-series clustering problem [36].

Two key challenges in time series clustering are defining similarity and selecting an appropriate clustering algorithm. We defined the similarity of users’ day-to-day travel profiles by comparing the number of trips at each time step, categorizing this as a ‘similarity in time’ objective. Therefore, Euclidean distance is a suitable measure of similarity and is used in this study. As for algorithm selection, efficiently handling high-dimensional, extensive time-series data requires clustering methods with low computational overhead. In this context, we applied the Balanced Iterative Reducing and Clustering using Hierarchies (BIRCH) algorithm for the second user segmentation. BIRCH is a hierarchical clustering method designed to efficiently cluster large datasets by first creating a concise summary of the data while preserving essential information. The BIRCH algorithm follows four key steps: 1) construct the CF-Tree by scanning the dataset; 2) compress the CF-Tree into a smaller representation; 3) perform global clustering; and 4) refine the clusters.

The number of clusters in the global clustering step is determined using a dendrogram, and the two critical parameters—threshold and branching factor—are optimized via the grid search method. The clustering results are then visualized using a matrix plot, illustrating the day-to-day activities of bus user segments during the outbreak period. Finally, the travel patterns of each segment are discussed in terms of temporal and spatial distribution.

## Results

### Travel patterns in the near-normal periods

**Fig 4:**
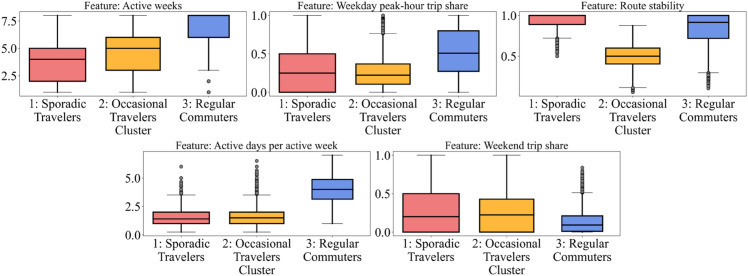
The distribution of cluster features of bus users in each travel pattern cluster.

To understand the diversity of travel patterns in the near-normal periods of the pandemic, we clustered all the observations of bus users by the travel behavior vector. The within-cluster sum of square distance and the Silhouette index are employed together to determine the appropriate number of clusters, which maximizes its value when there are three clusters. We numbered the clusters in ascending order of *Active weeks* and display the distribution of cluster features using box graphs to facilitate the interpretation of clustering results (See Fig 4). A name is assigned to each cluster based on its characteristics to aid in easier referencing.

Cluster 1 corresponds to the travel pattern segment of ‘Sporadic Travelers’. It is the largest cluster, accounting for 37.13% of feature vectors. This cluster is characterized by the lowest *Active weeks* and *Active days per active week*, indicating that users in this segment traveled only once or twice during a few active weeks. Due to the low travel frequency, the temporal and spatial characteristics are irregular. The distributions of the *Weekday peak-hour trip share* and *Weekend trip share* are highly varied, and the *Route stability* is closest to 1. This suggests that users in this segment rely on bus transit only occasionally, likely for specific purposes such as medical visits, family gatherings, shopping, or leisure activities. Therefore, their travel pattern indicates a low dependency on bus transit.

Cluster 2 represents the ‘Occasional Travelers’ segment, accounting for 35.82% of feature vectors. Like Cluster 1, the *Active days per active week* is also low, but the *Active weeks* count is higher (approximately 5 weeks out of 8), reflecting a more regular travel pattern and stronger bus transit usage. As a result, the temporal and spatial distribution of trips shows clearer patterns, with approximately 25% of trips during weekday peak hours and approximately 50% on the most frequently used route. We posit that users in this group use bus transit for non-work purposes such as shopping and entertainment, as well as occasional business trips like attending conferences or client meetings.

Cluster 3 represents the ‘Regular Commuters’ segment. While this cluster is the smallest (27.04% of feature vectors), it reflects the most stable commuting behavior. The *Active weeks* in this cluster approaches eight, indicating that these users traveled by bus nearly every week during the analysis period. The *Active days per active week* is around five, aligning with the number of weekdays, and most trips occur during weekday peak hours rather than weekends, confirming commuting patterns. The *Route stability* is also higher than in Cluster 2, as would be anticipated given the less variable nature of commuting routes compared to leisure or business trips.

Kruskal-Wallis tests are conducted on the five variables to determine whether the distributions of these variables present significant differences across the three clusters. The results are shown in Table 2. For all variables, the test statistics are highly significant with p-values of 0.000, indicating that there are statistically significant differences in the distributions of these variables across the groups being compared. This suggests that the K-means++ clustering method effectively captures the heterogeneity of travel behavior patterns among bus users in the Jiading bus system during the near-normal periods of the pandemic.

**Table 2:** Kruskal-Wallis test results of features in the three clusters.

Feature	H-value	p-value
*Active weeks*	162939.503	0.000
*Active days per active week*	250857.049	0.000
*Weekday peak-hour trip share*	58338.181	0.000
*Weekend trip share*	18425.441	0.000
*Route stability*	317918.870	0.000

### Changes in travel patterns after the outbreak period

**Fig 5:**
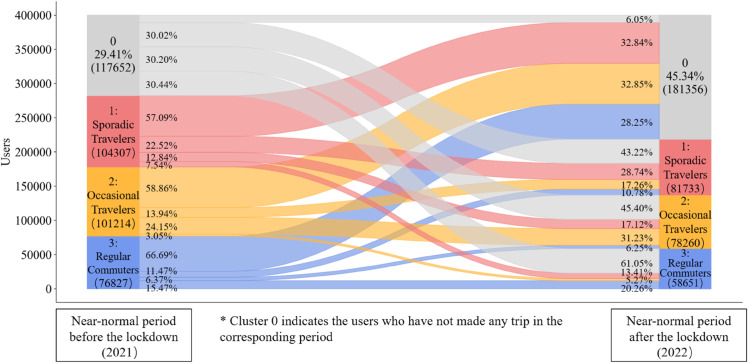
Travel patterns of Jiading bus users in the pre-outbreak and post-outbreak periods.

To analyze the heterogeneous changes in the travel patterns of different user segments after the outbreak, we compared the travel patterns of the same users in different periods using a Sankey diagram (see Fig 5). In this way, the number of bus users who have changed their travel patterns after experiencing the lockdown and how their travel patterns have been altered can be easily observed and quantified. The rectangles on the left represent the users with their travel patterns in the pre-outbreak period, while those on the right represent the post-outbreak period.

At a macro level, only 17.7% of users maintained their travel patterns. The Jiading bus system saw a 22.6% decline in active users, dropping from 282,348 to 218,644 after the outbreak. Although Regular Commuters accounted for a smaller proportion of those who discontinued bus usage (28.25%) compared to Sporadic Travelers (32.84%) and Occasional Travelers (32.85%), this segment exhibited the highest rate of bus usage discontinuation (66.69%). Our findings indicate that regular commuters exhibited the highest propensity to discontinue bus usage post-lockdown, possibly due to the shift to remote work or the adoption of alternative transport modes, such as private vehicles and micro-mobility.

When examining user flows between segments, we identified the main transition patterns. ‘Sporadic Travelers’ were most likely to become ‘Occasional Travelers’ after the lockdown, and vice versa. A significant portion of ‘Regular Commuters’ shifted to these two categories, indicating a reduction in bus use for commuting. This shows that non-commuters are unlikely to shift to regular bus use, while the opposite—commuters reducing their trips—is more common during the pandemic. In line with the macro analysis, ‘Regular Commuters’ represent the most vulnerable group during the pandemic, highlighting the need for planners and transit operators to focus on commuter travel behavior and demand during ridership crises.

An interesting pattern is the large number of users who did not travel by bus before the outbreak but started doing so afterward. While counter-intuitive amid overall user loss, this can be explained by the influx of migrants to Shanghai, whose numbers in some areas, including Jiading, surpass the local population (1,093,800 migrants vs. 853,600 locals) [37]. These new users likely include people who moved to Shanghai in 2022 and were not present in 2021. Other factors, such as newly enrolled students or young adults starting their first jobs, also contribute to this growth. Conversely, the 22.6% of lost users likely include both migrants who left the city and locals who stopped using the bus. Further sociodemographic data is needed to distinguish between these two groups and quantify their respective shares.

### Heterogeneity of day-to-day travel profiles during the outbreak period

The second step complements the analysis by inferring user attributes within various sub-segments based on the clustering of diverse day-to-day travel profiles observed during the outbreak period. According to the results in the first step, the ‘Sporadic Travelers’ traveled at a low frequency with irregular travel patterns in the near-normal patterns. Hence, these users are not likely to exhibit regular travel behavior in the outbreak period when the policy responses restricting mobility are far more intense. On the contrary, the ‘Occasional Travelers’ and ‘Regular Commuters’, especially those who did not change their travel patterns after the outbreak are attracting more attention, as they stand a good chance of belonging to the transit-dependent users. Therefore, we only included the users who belonged to Cluster 2 or Cluster 3 in the pre-outbreak period and did not change their travel patterns after the outbreak as the objects of the second step. The number of the included users is 34784, accounting for 8.70% of the total users in the Jiading bus system.

**Fig 6:**
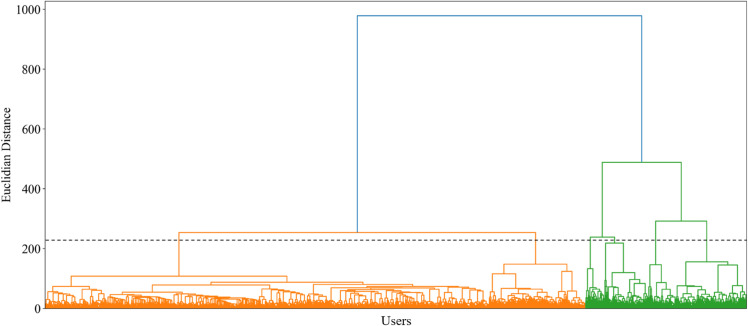
The dendrogram for the cluster centers of day-to-day travel profiles.

The dendrogram for the cluster centers of day-to-day travel profiles is shown in Fig 6 to determine the number of clusters in the global clustering step in the BIRCH algorithm. After testing with various cluster specifications, we discovered that 6 clusters and a parameter setting with *threshold* = 0.1 and *branchingfactor* = 10 exhibit compact clusters with significant differences in day-to-day travel profiles between clusters.

**Fig 7:**
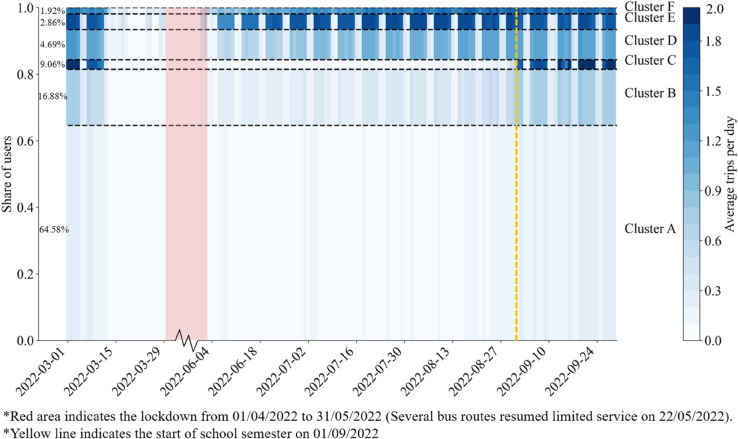
The day-to-day travel profile of ‘Occasional Travelers’ and ‘Regular Commuters’ in different sub-segments.

Fig 7 provides the day-to-day travel profiles of bus users in the 6 clusters, where the darkness of colors in the matrices indicates the average number of trips conducted by the users, and the heights represent the proportion of users in the clusters. Due to the strict policy responses, especially the two-month citywide lockdown, users in the second cluster exited the bus system before the lockdown and returned after it was lifted. Key differences between user segments relate to the average daily trips and the timing of leaving and returning.

Among the 6 clusters, Cluster A is the only user sub-segment characterized by the low usage of bus transit from beginning to end. Pre-lockdown, users averaged 0.1 to 0.2 trips per day, meaning only 10% to 20% of users traveled on a given day. Despite low usage, they were sensitive to the pandemic, reducing travel as early as March, with a slow recovery lasting until late September. Most (91.62%) of these users are ‘Occasional Travelers’, whose patterns remained stable in the first step.

Clusters B and C saw a significant increase in trips when the new school semester began, suggesting these users are students, teachers, or parents. Both groups traveled more on weekdays but differed in daily trip frequency and timing of return. Cluster B users averaged one trip per weekday post-outbreak, while Cluster C averaged two, showing more regularity. Both exited in mid-March, but Cluster B returned quickly by June, despite fewer trips, whereas Cluster C didn’t return until September. Cluster B users are likely parents, with weaker bus dependency, possibly owning cars or micro-mobility vehicles, as their usage is not enough for a full commute. Cluster C users, likely students, show higher bus dependency, as they rarely use buses during school breaks.

Clusters D to F exhibited regular daily travel patterns, characterized by late departures until late March and early returns starting in late May. Intuitively, this timing pattern appears to be independent of school schedules, suggesting that these users are likely commuters using buses for work rather than for school. In light of the policy context, where only ‘essential workers’ employed in designated enterprises were allowed to return to work between May 22 and May 31, 2022, many users in these clusters are likely to belong to this group.

Despite these overall similarities, there are two key distinctions between the three clusters. First, there is a difference in weekend travel patterns. While Clusters D and E mainly travel on weekdays, Cluster F stands out with steady travel throughout the week including weekends, which may be due to non-commuting activities such as shopping or leisure, or jobs with irregular hours requiring weekend travel. Further analysis of trip timing and locations is necessary to confirm the attributes of these users. The second distinction lies in the average number of daily trips between Clusters D and E. Cluster D users make more trips on average, while Cluster E users make fewer, suggesting varying levels of bus dependence. Users in Cluster D, with lower dependence, may have access to other modes of transport, such as personal vehicles or micro-mobility options, while users in Cluster E are more reliant on buses.

To further validate the inferred characteristics of bus users across the six sub-segments, we analyzed the temporal and spatial patterns of their trips, represented by trip start times and route types. Since complete data on boarding and alighting stops was unavailable, we categorized the Jiading bus network routes into four types based on the start and end stop locations (see Table 3), linking spatial patterns to route types.

**Table 3:** The division principle of four types of routes in Jiading bus system.

Type of routes	Definition	Types of trips served
Urban routes	The routes with both ends located within the urban area of Jiading district	Short-distance, within the urban area
Suburban routes	The routes with both ends located within the same satellite town	Short-distance, within a certain satellite town
Regional routes	The routes that have one end within the urban area and the other in satellite towns, or have both ends in different satellite towns	Long-distance, between different regions in the Jiading district
Inter-district routes	The routes with one end located within Jiading District and the other outside	Long-distance, between Jiading district and other districts

The rationale for this division is supported by one-day trajectory data from Alipay, which uses payment records—covering both bus travel and other consumption scenarios—to infer the boarding and alighting stops of each trip within the Jiading bus network on September 10, 2021. From this data, we can estimate the average travel distance for each bus route. The results indicate that, for all Urban and Suburban routes, the median of average travel distances are 3.7 km and 4.3 km, respectively, both lower than the distances for Regional routes (5.5 km) and Inter-district routes (7.7 km). To test for statistical significance, Mann-Whitney U tests were performed across the different route types. The average travel distance of Inter-district routes was found to be significantly higher than that of Urban routes (U=9.5, p=0.002) and Suburban routes (U=9.68, p=0.001). Similarly, the differences between Regional and Urban routes (U=7.70, p=0.005) as well as Regional and Suburban routes (U=3.96, p=0.046) were also significant. This reinforces the validity of dividing travel distances based on route types.

**Fig 8:**
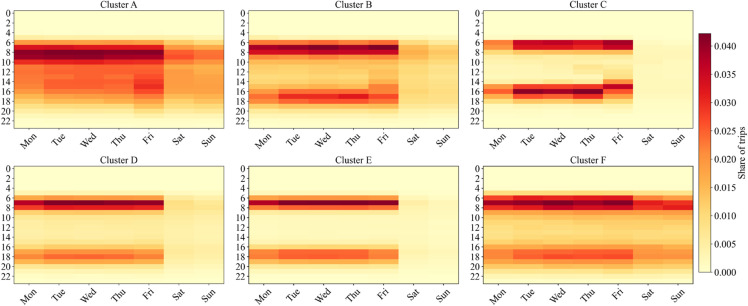
The distribution of start times of trips conducted by users in each sub-segment.

**Fig 9:**
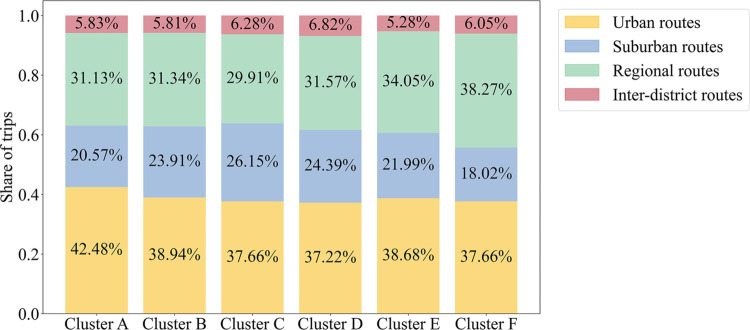
The distribution of trips over different types of routes in each sub-segment.

Fig 8 shows the distribution of trips across different times and days of the week, while Fig 9 presents the distribution across route types. Cluster A users primarily begin trips on weekday mornings, with the rest of their trips evenly spread across various hours and days. For the other five sub-segments, trips are concentrated during morning and evening peak hours. These time patterns align with the expected travel patterns during near-normal periods.

However, differences exist in the specific peak times and concentration levels. Clusters B and C have morning peaks from 7 to 8 a.m. and evening peaks from 3 to 5 p.m. On Fridays, evening peak travel occurs earlier than Monday through Thursday, aligning with the schedules of primary and secondary schools in Shanghai. This confirms that Clusters B and C largely consist of students and school-related travelers. Additionally, Cluster B shows some off-peak weekday and holiday trips, likely reflecting parents combining school pick-ups with other activities like shopping or errands.

Clusters D to F, representing commuters, show distinct temporal patterns. Their morning peaks are also between 7 and 8 a.m., but evening peaks are later, from 5 to 6 p.m., compared to the earlier peaks of students and parents. Cluster F also shows strong weekend peaks, indicating workers on irregular schedules. The few trips during peak hours may also reflect non-work-related travel.

The spatial distribution of trips by route type further supports the inferred user attributes. Based on the route categories in Table 2, we assume that the proportion of short-distance trips within the region corresponds to the proportion of trips on Urban and Suburban routes. Among the six clusters, Cluster C exhibits the highest proportion of within-region trips (63.81%), reflecting the tendency of most primary and secondary school students in Shanghai to attend nearby schools. Additionally, from Cluster D to Cluster F, the average daily trips increase while the proportion of within-region trips declines. This fits the expectation that short trips are more likely to be replaced by alternatives like micro-mobility, while longer, inter-regional trips depend more on bus transit.

## Conclusions

This study presents a data-driven study conducted in Shanghai, China, examining behavioral patterns among individuals during and after the lockdown period. Our analysis identifies distinct groups based on whether they maintained or altered their travel patterns, while incorporating insights from policy response measures. Through a two-step analytical framework, we uncover several key findings that address the two research questions.

For the first research question, our analysis reveals that Jiading bus system users during near-normal periods fall into three distinct segments: ‘Sporadic Travelers’, ‘Occasional Travelers’, and ‘Regular Commuters’. The lockdown disproportionately impacted ‘Regular Commuters’, who exhibited a higher likelihood of either shifting to non-commuting travel patterns or permanently ceasing bus usage in the post-lockdown stage. Notably, only 17.7% of bus users retained their pre-lockdown travel habits in the post-pandemic period.

For the second research question, we identify six sub-segments among those who maintained their travel patterns, each characterized by distinct day-to-day travel profiles. By examining exit and return times in conjunction with policy responses, we deduce that these sub-segments include school students, their parents, and employees with varying work schedules and bus transit dependencies. These findings are further substantiated by the spatial and temporal distribution of trips.

Our findings demonstrate both similarities and differences when compared with international cases. Regarding changes in travel patterns, we observe a global trend toward less regular transit use, consistent with findings from Montreal where telecommuting has led to more occasional public transit usage [38]. However, the extent of this behavioral shift varies considerably across different contexts. In Jeju, Korea—where only mild interventions like social distancing were implemented without major outbreaks—nearly half of users maintained their original travel frequency, with just one-third reducing usage [28]. This contrasts sharply with our Jiading case study, where approximately 60% of users transitioned to lower-frequency travel patterns, indicating a more substantial behavioral change (Fig 5). When examining user attributes, our results partially align with Stockholm’s findings regarding socioeconomic factors [10], as we similarly observed that bus-dependent users in Clusters D through F—typically representing lower-income groups—were more likely to maintain their public transit usage despite changing circumstances.

The findings offer useful guidance for PT operators and governments in future public health crises. First, special attention should be given to ‘Regular Commuters’, who seem most vulnerable to pandemic-related disruptions. PT operators could introduce adaptive services and incentives to retain these users, such as flexible fare structures or tailored routes. Understanding their reasons for permanently discontinue bus usage, possibly through surveys, could help refine these strategies. For example, if safety and health concerns are a major factor, operators should ensure a clean and safe environment to restore passengers’ confidence. Second, comprehensive measures are needed to revitalize bus transit in the wake of significant shifts in travel behavior post-pandemic. Governments should recognize the critical role of PT in urban mobility and economic recovery and consider offering financial support to PT operators. Additionally, flexible regulatory frameworks are essential to allow rapid adjustments to PT services in response to changing travel demands. This could include streamlined processes for route changes, fare adjustments, and safety measures.

This study has two primary limitations. First, the absence of sociodemographic data in the ticketing system prevents validation of user identities across the identified segments. Future research could mitigate this gap by incorporating targeted sampling surveys to collect supplementary sociodemographic information. Second, while we identify shifts in travel patterns, our analysis does not quantitatively assess their underlying causes. Subsequent studies should integrate variables such as individual exposure levels, sociodemographic characteristics, and built environment factors to develop a robust explanatory model for these behavioral changes.
